# SUCCESSFUL AGING AND HEALTHCARE SERVICE UTILIZATION IN EAST ASIA: NATIONAL COMPARISONS OF CHINA, KOREA, AND JAPAN

**DOI:** 10.1093/geroni/igz038.2647

**Published:** 2019-11-08

**Authors:** Jinmyoung Cho, Takeshi Nakagawa, Dannii Yeung

**Affiliations:** 1 Baylor Scott & White Health, Temple, Texas, United States; 2 National Center for Geriatrics and Gerontology, Aichi, Japan; 3 City University of Hong Kong, Hong Kong, Hong Kong

## Abstract

As the older population increases and lives longer, the demand for healthcare has been increased dramatically. To date, it is unknown whether older people’s healthcare utilization varies between countries and how it relates to successful aging. Using Rowe and Kahn’s model, we examine cross-national differences in the relationship between successful aging and healthcare service utilization in East Asia. Harmonized datasets at baseline from China Health and Retirement Longitudinal Study (CHARLS), Korean Longitudinal Study (KLoSA), and Japanese Study on Aging and Retirement (JSTAR) were used. Including 7,651 participants (aged 65-75 years), successful agers were identified using Rowe and Kahn’s criteria (i.e., no disease, no disability, high cognitive function, and active engagement). Healthcare service utilization includes hospital visit, number of hospital stay, number of nights per hospital stay, regular medical center visit, number of medical center visits, and possession of private insurance in previous year. Generalized linear models showed that successful agers’ healthcare service utilization is significantly different from non-successful agers (e.g., OR=2.19, p<.001 for regular medical center visits), and Korean and Chinese healthcare service utilization is different from Japanese (e.g., OR=0.44 and OR=10.18 for Chinese and Korean number of medical center regular visits, respectively, p<.001). Furthermore, the number of nights in hospital among Chinese and Korean successful agers tend to be greater than that of Japanese successful agers (OR=2.93 and OR=1.99 for Chinese and Korean successful agers, respectively, p<.001). This study indicates cross-national variations in pattern of healthcare service utilization between successful and non-successful agers in East Asia.

